# Supplementary material for: 40 Hz sensory stimulation enhances CA3-CA1 coordination and prospective coding during navigation in a mouse model of Alzheimer’s disease
Source: bioRxiv. 2024 Oct 23:2024.10.23.619408. Preprint. [Version 1] doi: 10.1101/2024.10.23.619408 (PMC11526945; doi:10.1101/2024.10.23.619408)
Supplement: Supplement 1 [file media-1.pdf]

Supplementary Materials for

40 Hz sensory stimulation enhances CA3-CA1 coordination and prospective coding during navigation in a mouse model of Alzheimer's disease

Abigail L. Paulson, Lu Zhang, Ashley M. Prichard, Annabelle C. Singer

Supplement Includes:

- Supplementary Figures 1-10
- Supplementary Tables 1-6

Supplementary Figures

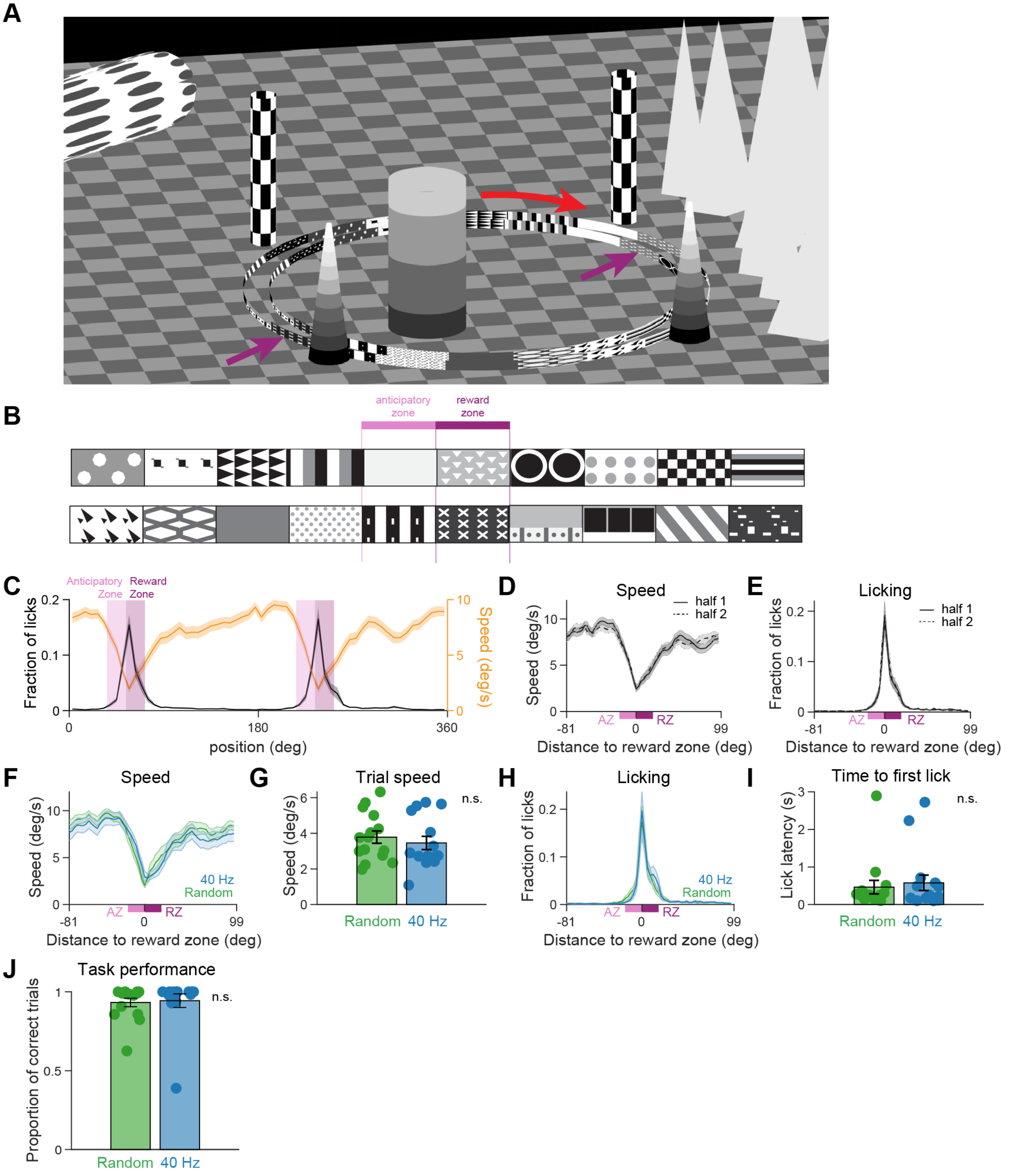

Supplementary Figure 1. 5XFAD mice perform similarly in a goal-directed spatial navigation task after 8 days of 40 Hz or Random stimulation.

- **A.** Overhead view of the virtual reality environment. The mouse runs through the annular track in the direction indicated by the red arrow. The reward zones are indicated with purple arrows

(arrows are not part of the environment and are not shown to the animal). Distal cues (pillars, floating objects) are visible above the patterned walls as the mouse runs through the track.

B. Detailed schematic of the patterned cues in the track centered around the anticipatory and reward zones. Each patterned cue covers 18 degrees of the 360 track.

C. Average speed (orange, right y-axis) and licking (black, left y-axis) as a function of position in the annular environment. 360 degrees indicates a full traversal of the track. Reward zones, located 180 degrees apart, are indicated by dark pink shaded areas with anticipatory zones just before reward zones indicated by light pink shaded areas. Mean ± SEM across recording days. Random, n = 15 days in 8 mice; 40 Hz, n = 15 days in 8 mice.

D. Speed as a function of position is similar between the two halves of the track (solid line, first half and dashed line, second half). Animals display similar slowing behavior in the anticipatory zone (AZ, light pink) as they approach both reward zones (RZ, dark pink). Mean ± SEM across recording days. Random, n = 15 days in 8 mice; 40 Hz, n = 15 days in 8 mice.

E. As animals approach the rewarded areas of the track, licking increases in anticipation of the reward. When the animals are in the reward zone (dark pink), increased licking indicates reward consumption. Anticipatory licking behavior occurs before both reward zones in the anticipatory zone (light pink). Mean ± SEM across recording days. Random, n = 15 days in 8 mice; 40 Hz, n = 15 days in 8 mice.

F. Animals exposed to 40 Hz (blue) and Random (green) flicker show anticipatory slowing behavior as they approach the reward zone. Mean ± SEM across recording days. Random, n = 15 days in 8 mice; 40 Hz, n = 15 days in 8 mice.

G. Average trial speed per day for animals exposed to 40 Hz (blue) and Random (green) flicker. Mean ± SEM across recording days. 40 Hz, n = 15 days in 8 mice; Random, n = 15 days in 8 mice, P=0.51, n.s., linear mixed-effects model (LME). See Supplementary Table 3 for statistical details.

H. As in **D** for licking behavior. Animals exposed to 40 Hz and Random flicker show anticipatory licking behavior as they approach the reward zone. Random, n = 15 days in 8 mice; 40 Hz, n = 15 days in 8 mice. Mean ± SEM across recording days.

I. Average time to first lick in the rewarded zones of the track. Most animals lick quickly (<1 s) following entry into the reward zone. Reward is not delivered until animals lick inside the rewarded zone. Mean ± SEM across recording days. 40 Hz, n = 15 days in 8 mice; Random, n = 15 days in 8 mice, P=0.74, n.s., LME.

J. Task performance was not significantly different between animals exposed to 40 Hz or Random flicker. Mean ± SEM across recording days. 40 Hz, n = 15 days in 8 mice; Random, n = 15 days in 8 mice, P=0.85, n.s., LME.

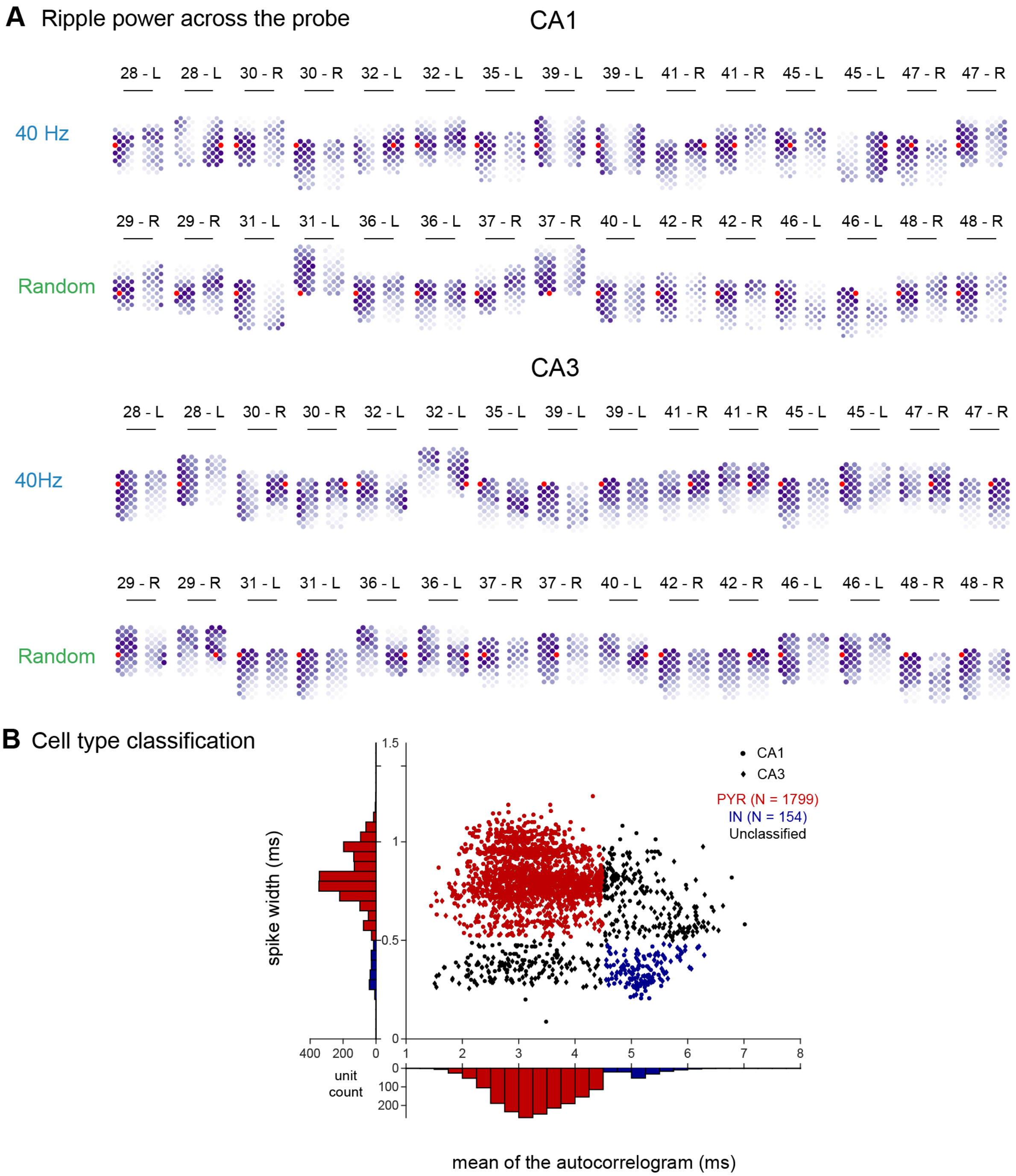

Supplementary Figure 2. Recording locations and cell type classification.

A. Recording location for all recording sessions in CA1 (top two rows) and CA3 (bottom two rows) is shown by quantifying the ripple power (150-250 Hz) across the probe. The electrode has two shanks, each with 32 channels. Each circle is a recording channel. The color of the channel corresponds to the ripple power recorded on the channel; darker purple colors indicate higher ripple power with the red channel indicating the largest ripple power channel on each recorded day (used for ripple analyses, see Methods). The ripple power is expected to be highest in the center of the pyramidal cell layer. Probe diagrams are aligned to the channel with the highest

ripple power. The animal ID and recording hemisphere (left (L) or right (R)) are noted above each probe illustration. Two recording sessions were performed from each hemisphere.

B. Recorded single units were classified into putative pyramidal cells (red) and putative interneurons (blue) based on spike width and the mean of the autocorrelogram. The same criteria were applied to CA1 units (circles) and CA3 units (diamonds). Unclassified cells are marked in black and were excluded from further analyses. CA1, n = 1011 pyramidal cells, n = 54 interneurons; CA3, n = 788 pyramidal cells, n = 100 interneurons.

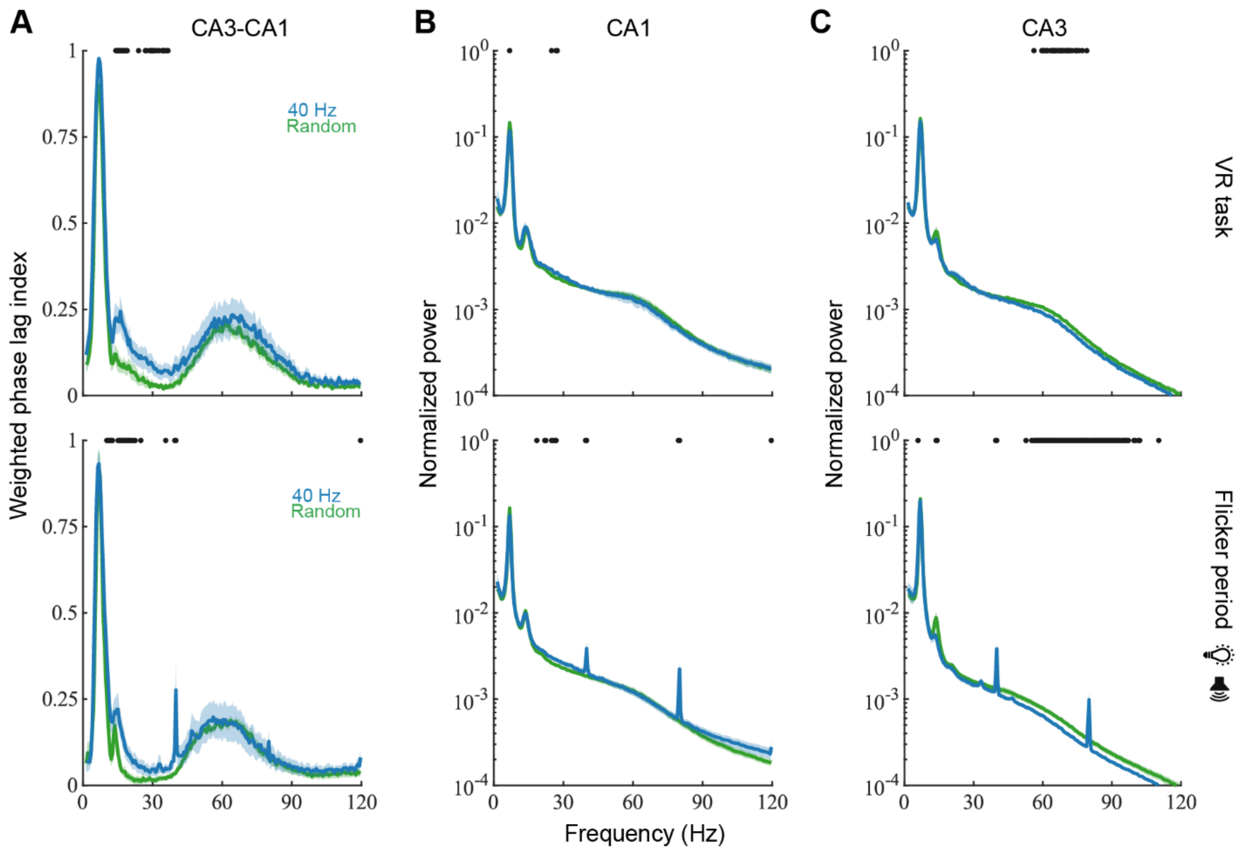

Supplementary Figure 3. CA3-CA1 LFP phase synchrony is enhanced in the slow gamma band following 40 Hz flicker.

- **A.** Weighted phase lag index between LFPs recorded in CA3 and CA1 during VR navigation periods (top) and flicker stimulation periods (bottom) after exposure to 40 Hz (blue) or Random (green) flicker. Weighted phase lag index measures functional connectivity with minimal volume conduction effects. Functional connectivity between CA3 and CA1 was higher after 40 Hz than Random flicker in slow gamma bands. Dots above the graph indicate frequencies with significant differences between 40 Hz and Random flicker exposure groups. $p < 0.05$, unpaired t-test without correction, 40 Hz, n = 15 days in 8 mice; Random, n = 15 days in 8 mice. See Supplementary Table 4 for statistical details. Mean $\pm$ SEM across recording days.
- **B.** Normalized power spectral density of LFPs recorded in CA1 during VR navigation task periods (top) and flicker stimulation periods (bottom) after exposure to 40 Hz (blue) or Random (green) flicker. Dots indicate frequency points with significant differences between 40 Hz and Random ($p < 0.05$, unpaired t-test without correction, 40 Hz, n = 15 days in 8 mice; Random, n = 15 days in 8 mice). Mean $\pm$ SEM across recording days.
- **C.** Same as **B** for LFPs in CA3.

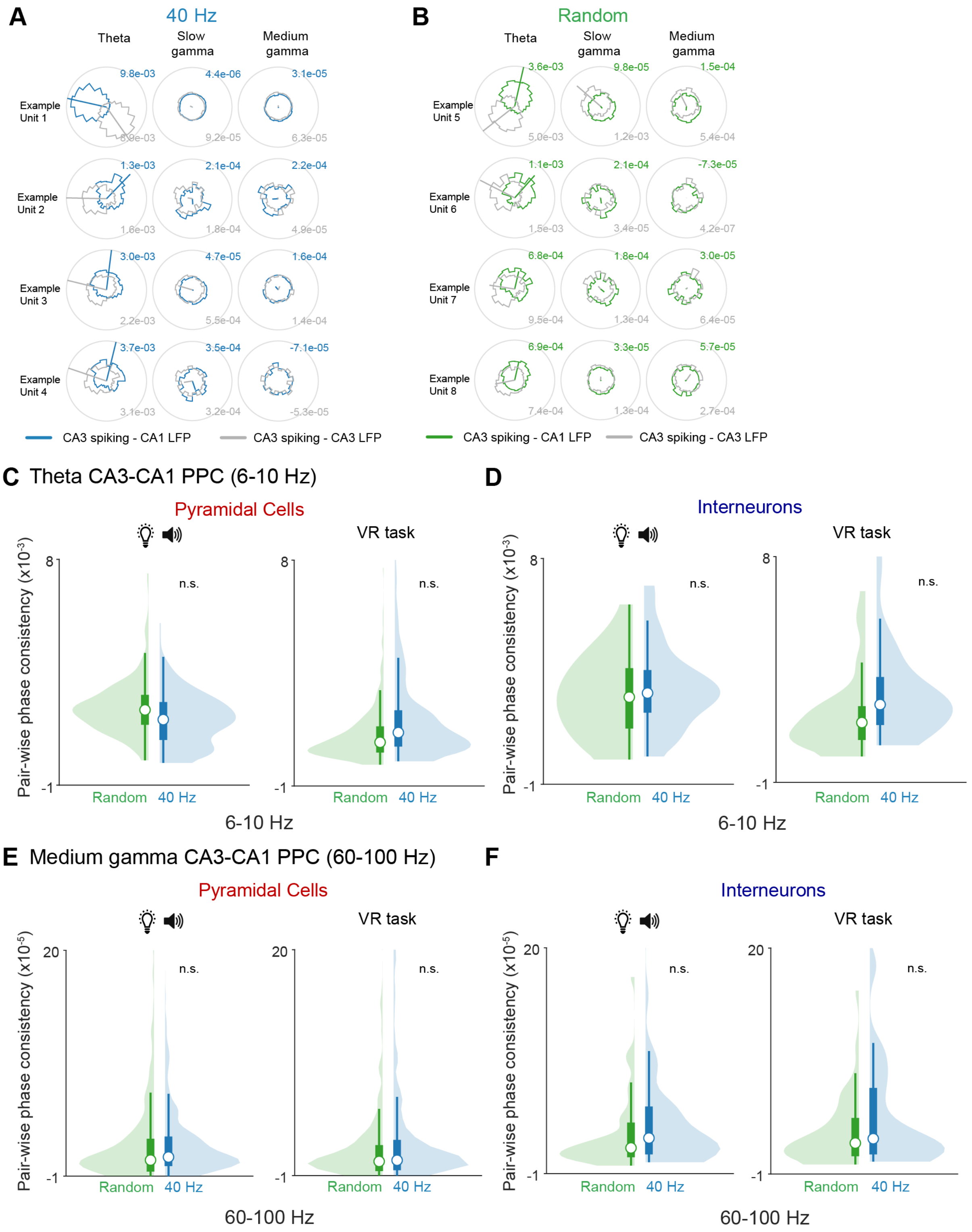

Supplementary Figure 4. Theta and medium gamma PPC are similar following 40 Hz or Random flicker.

A. Local (grey) and cross-region (blue) spike-phase coupling for example single units in CA3 and LFPs in CA1 during periods of theta (left), slow gamma (center), and medium gamma (right) after exposure to 40 Hz flicker. Each row is an example single unit.

B. Same as **A** for example CA3 single units from animals exposed to Random stimulation with local coupling in grey and cross-region coupling in green.

C. Theta (6-10 Hz) spike-field pairwise phase consistency (PPC) for putative CA3 pyramidal cells and CA1 LFPs during flicker stimulation periods (left) and during VR behavior periods (right) following 8 days of exposure to 40 Hz (blue) or Random (green) flicker stimulation. *Left*, flicker stimulation periods, Random, n = 320 pyramidal cells from 8 mice; 40 Hz n = 387 pyramidal cells from 8 mice; P = 0.19, n.s., linear mixed-effects model (LME). *Right*, VR task periods, Random, n = 347 pyramidal cells from 8 mice; 40 Hz, n = 335 pyramidal cells from 8 mice; P = 0.29, n.s., LME. See Supplementary Table 3 for statistical details. Violin plots throughout include a histogram (shaded area) and box plot indicating median (white circle), first and third quartiles (dark box) and whiskers (thin lines).

D. As in **B** for putative CA3 interneurons. *Left*, flicker stimulation periods, Random, n = 46 interneurons from 8 mice; 40 Hz, n = 50 interneurons from 8 mice; P = 0.36, n.s., LME. *Right,* VR task periods, Random, n = 49 interneurons from 8 mice; 40 Hz, n = 50 interneurons from 8 mice; P = 0.12, n.s., LME.

E. Medium gamma (60-100 Hz) spike-field pairwise phase consistency (PPC) for putative CA3 pyramidal cells and CA1 LFPs during flicker stimulation periods (left) and during VR behavior periods (right) following 8 days of exposure to 40 Hz (blue) or Random (green) flicker stimulation. *Left*, flicker stimulation periods, Random, n = 320 pyramidal cells from 8 mice; 40 Hz n = 387 pyramidal cells from 8 mice; P = 0.90, n.s., linear mixed-effects model (LME). *Right*, VR task periods, Random, n = 347 pyramidal cells from 8 mice; 40 Hz, n = 335 pyramidal cells from 8 mice; P = 0.32, n.s., LME.

F. As in **E** for putative CA3 interneurons. *Left*, flicker stimulation periods, Random, n = 46 interneurons from 8 mice; 40 Hz, n = 50 interneurons from 8 mice; P = 0.21, n.s., LME. *Right,* VR task periods, Random, n = 49 interneurons from 8 mice; 40 Hz, n = 45 interneurons from 8 mice; P = 0.11, n.s., LME.

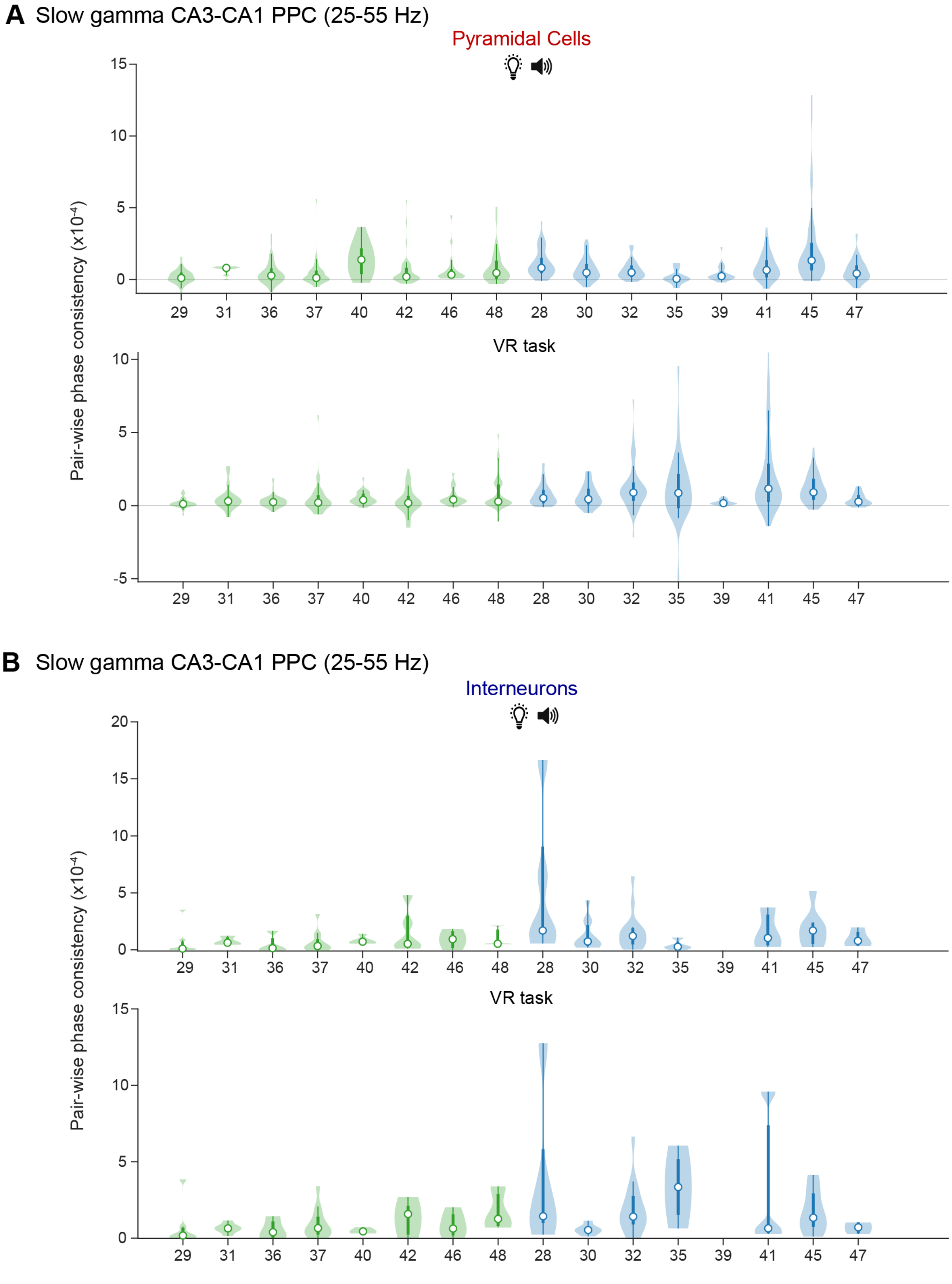

Supplementary Figure 5. Slow gamma CA3-CA1 pairwise phase consistency distributions per animal.

A. Slow gamma (25-55 Hz) pairwise phase consistency between spiking from putative CA3 pyramidal cells and CA1 LFP during flicker exposure (top) and VR behavior (bottom) periods per animal. Each violin plot contains data from one animal. The animal ID is noted on the x-axis.

B. Same as in **A** but for putative CA3 interneurons.

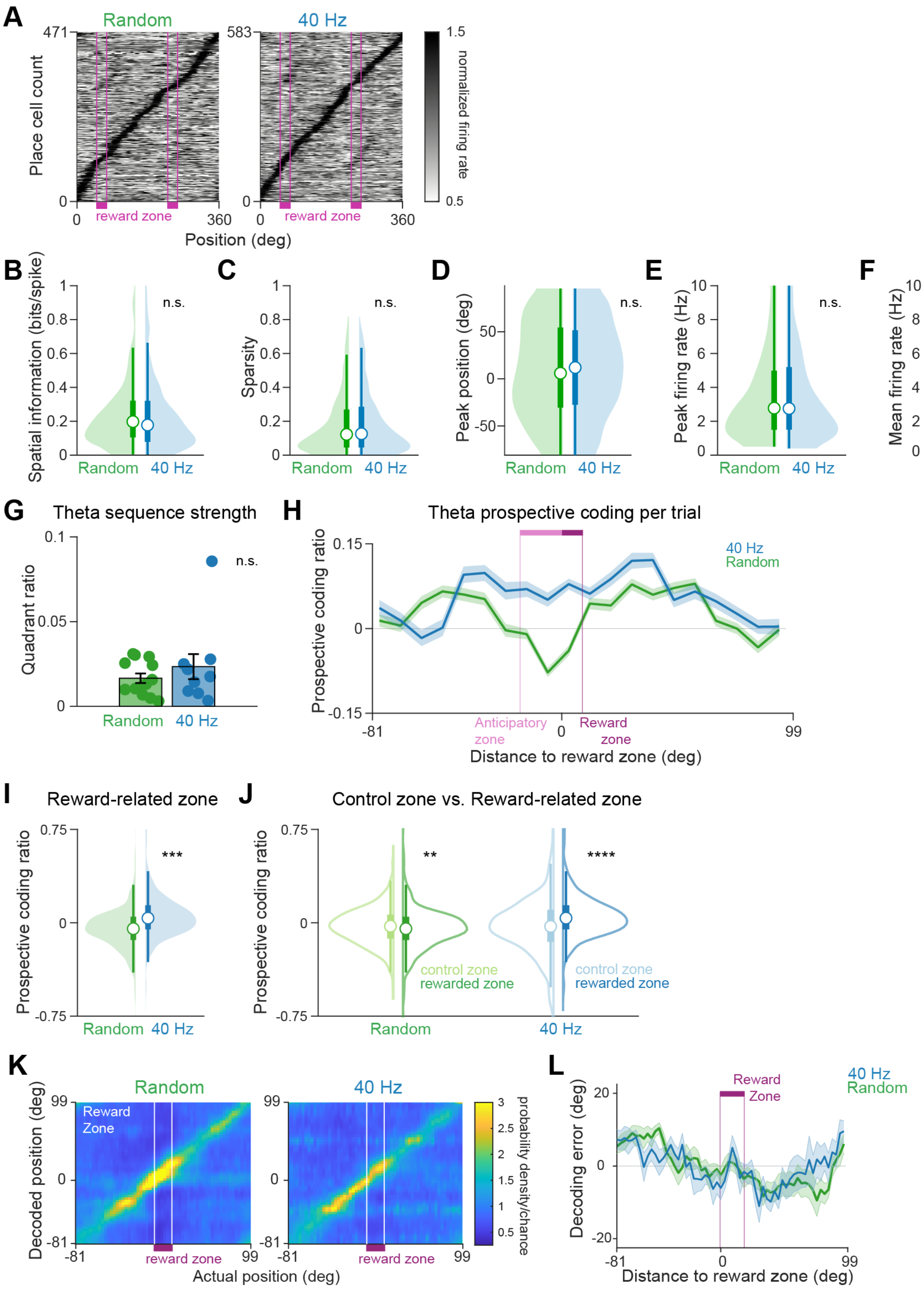

Supplementary Figure 6. Place cell properties and decoding performance are similar following 40 Hz and Random flicker.

- **A.** Normalized firing rate maps for place cells recorded from animals exposed to Random (left) or 40 Hz (right) stimulation with each row indicating a single place cell. Cells are ordered by peak firing

position in the track. Reward zones are indicated by pink lines and boxes along the x-axis. Random, n = 471 place cells from 8 mice; 40 Hz, n = 583 place cells from 8 mice.

B. Distribution of spatial information of place cells shown in **A** following 8 days of 40 Hz (blue) or Random (green) flicker exposure. Spatial information was computed from rate maps that were calculated as a function of distance to the reward zone in the track, as pictured in **Figure 3G**. Random, n = 471 place cells from 8 mice; 40 Hz, n = 583 place cells from 8 mice, P = 0.93, n.s.; linear mixed-effects model (LME). See Supplementary Table 3 for statistical details.

C. As in **B** for place cell sparsity, which ranges from 0 (the cell fires equally in all spatial bins) to 1 (the cell only fires in one spatial bin) to assess the relative proportion of the track in which the cell fired. Random, n = 471 place cells from 8 mice; 40 Hz, n = 583 place cells from 8 mice; P = 0.69, n.s., LME.

D. As in **B** for peak firing position of place cells as a function of distance to the reward zone. Random, n = 471 place cells from 8 mice; 40 Hz, n = 583 place cells from 8 mice; P = 0.68, n.s., LME.

E. As in **B** for maximum firing rate of place cells. Random, n = 471 place cells from 8 mice; 40 Hz, n = 583 place cells from 8 mice; P = 0.86, n.s., LME.

F. As in **B** for average firing rate of place cells. Random, n = 471 place cells from 8 mice; 40 Hz, n = 583 place cells from 8 mice; P = 0.85, n.s., LME.

G. Average theta sequence strength per day following 8 days of 40 Hz (blue) or Random (green) flicker exposure. Mean $\pm$ SEM across recording days. Random, n = 13 days from 8 mice; 40 Hz, n = 10 days from 6 mice; P = 0.37, n.s., LME.

H. Prospective coding ratio over position, calculated per trial. 8 days of 40 Hz flicker (blue) exposure lead to more prospective coding (positive values) in and around the anticipatory zone (light pink) and reward zone (dark pink) than Random flicker (green). Mean $\pm$ SEM across trials. Random, n = 934 trials from 8 mice; 40 Hz, n = 807 trials from 6 mice.

I. Per trial prospective coding ratio values in the anticipatory and reward zones were higher following 8 days of 40 Hz (blue) than Random (green) flicker exposure. Random, n = 934 trials from 8 mice; 40 Hz, n = 807 trials from 6 mice; P = 0.0007***, LME.

J. Prospective coding ratio was significantly higher after 40 Hz flicker in the reward-related areas (dark colors) of the track than a control location (light colors), where the animal did not receive reward. Random, control zone, n = 931 trials, reward-related zone, n = 934 trials, P = 0.009**, LME. 40 Hz, control zone, n = 807 trials, reward-related zone n = 799 trials, P = 1.4e-13****, LME.

K. Actual position of the animal versus Bayesian decoding of current spatial position based on neural activity, averaged across days shows strong decoding of current position following 8 days of 40 Hz (blue) or Random (green) flicker exposure. This validates our decoding approach. Warmer colors along the x=y diagonal indicate accurate neural decoding of the animal's current actual position. The reward zone is indicated by the white lines. Random, n = 13 days from 7 mice; 40 Hz, n = 13 days from 7 mice.

L. Decoding error between true and decoded position across the track, averaged across days. Values near zero indicate accurate neural decoding of the animal's current actual position. Decoding accuracy was high, especially approaching and in the reward zone (pink). Mean $\pm$ SEM across recording days. Random, n = 13 days from 7 mice; 40 Hz, n = 13 days from 7 mice.

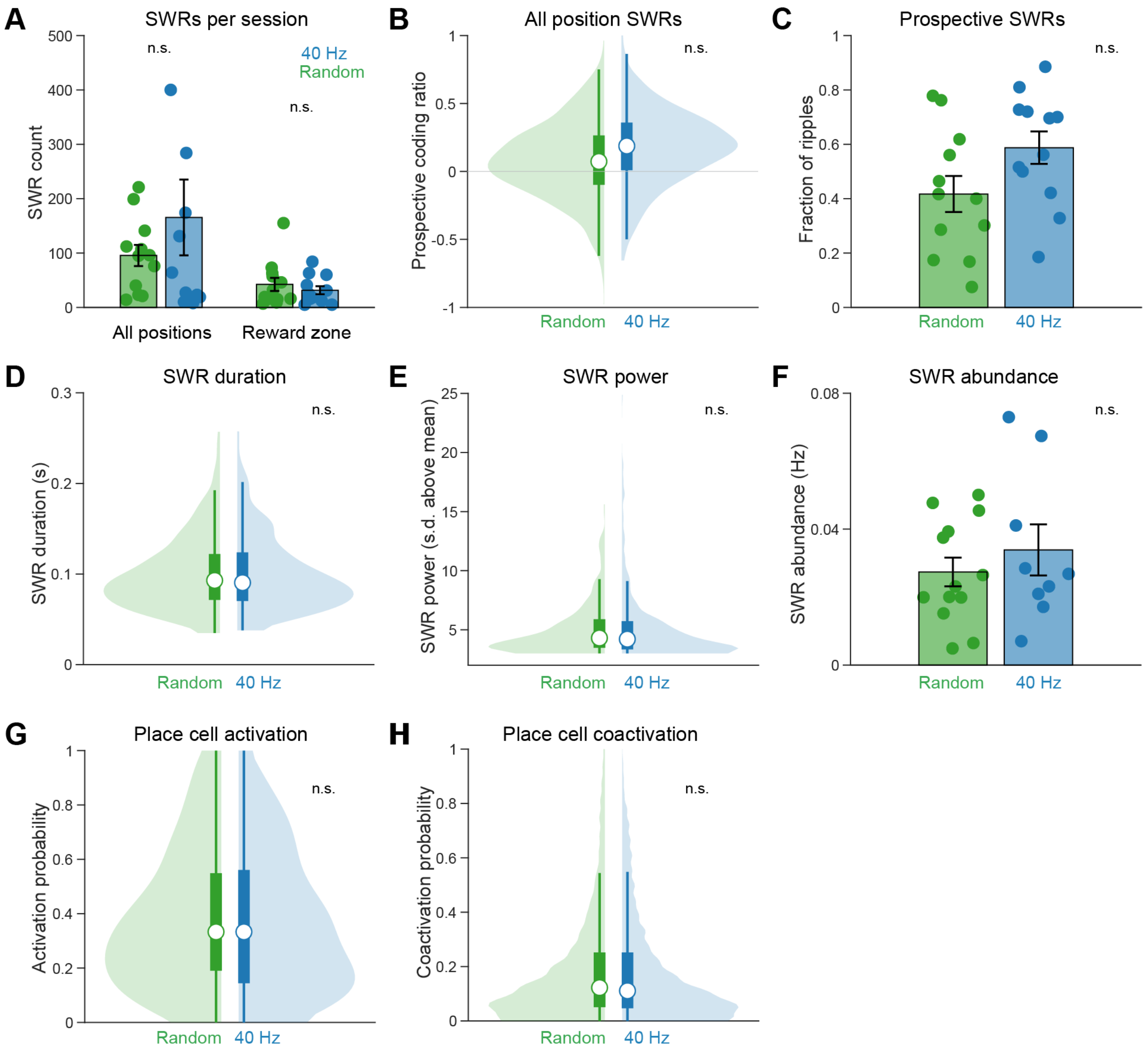

Supplementary Figure 7. Sharp-wave ripple properties are similar following 40 Hz and Random flicker.

- **A.** Number of SWRs detected per session for SWRs occurring at all track positions (left) and SWRs occurring inside of the reward zone (right) did not differ significantly following 8 days of 40 Hz (blue) or Random (green) flicker. Only sessions with more than 5 SWRs and with significant decoding (see Methods) were included. Mean ± SEM across recording days. *Left,* SWRs occurring in any position in the track, Random, n = 12 days from 7 mice; 40 Hz, n = 12 days from 7 mice; P = 0.46, n.s., linear mixed-effects mode (LME). *Right*, SWRs occurring in the reward zone of the track, Random, n = 12 days from 7 mice; 40 Hz, n = 12 days from 7 mice; n.s., LME. See Supplementary Table 3 for statistical details.
- **B.** Distribution of prospective coding ratio values for all SWRs occurring at any position in the track did not differ significantly following 8 days of 40 Hz (blue) or Random (green) flicker, unlike SWRs in the reward zone (**Fig. 4C**). Random, n = 1145 SWRs from 7 mice; 40 Hz, n = 1989 SWRs from 7 mice; P = 0.21, n.s., LME.
- **C.** Related to **B,** fraction of SWRs occurring in any location with prospective coding ratios greater than 0.1 did not differ significantly following 8 days of 40 Hz (blue) or Random (green) flicker,

unlike SWRs in the reward zone (**Fig. 4E**). Mean ± SEM across recording days. Each dot indicates one recording session. Random, n = 12 days from 7 mice; 40 Hz, n = 12 days from 7 mice; P = 0.12, n.s., LME.

D. Sharp-wave ripple duration for SWRs occurring inside the reward zone did not differ significantly following 8 days of 40 Hz (blue) or Random (green) flicker. Random, n = 572 SWRs from 8 mice; 40 Hz, n = 475 SWRs from 8 mice; P = 0.75, n.s., LME.

E. Sharp-wave ripple power for SWRs occurring inside the reward zone. Random, n = 572 SWRs from 8 mice; 40 Hz, n = 475 SWRs from 8 mice; P = 0.71, n.s., LME.

F. Average SWR abundance in the reward zone did not differ significantly following 8 days of 40 Hz (blue) or Random (green) flicker. SWR abundance was calculated during times in the reward zone where speed was slower than 1 deg/s. Only days with greater than 5 SWRs that met the speed criteria were included. Each dot indicates one recording session. Mean ± SEM across recording days. Random, n = 13 days from 8 mice; 40 Hz, n = 9 days from 6 mice; P = 0.45, n.s., LME.

G. Activation probability of place cells in CA1 and CA3 during SWRs occurring inside the reward zone did not differ significantly following 8 days of 40 Hz (blue) or Random (green) flicker. Place cells from sessions with greater than 10 SWRs in the reward zone were included. Random, n = 341 place cells from 7 mice; 40 Hz, n = 426 place cells from 7 mice; P = 0.78, n.s., LME.

H. Coactivation probability of all place cell pairs during SWRs occurring inside the reward zone did not differ significantly following 8 days of 40 Hz (blue) or Random (green) flicker. Place cell pairs from sessions with greater than 10 SWRs in the reward zone were included. Random, n = 6519 place cell pairs from 7 mice; 40 Hz, n = 9641 place cell pairs from 7 mice; P = 0.89, n.s., LME.

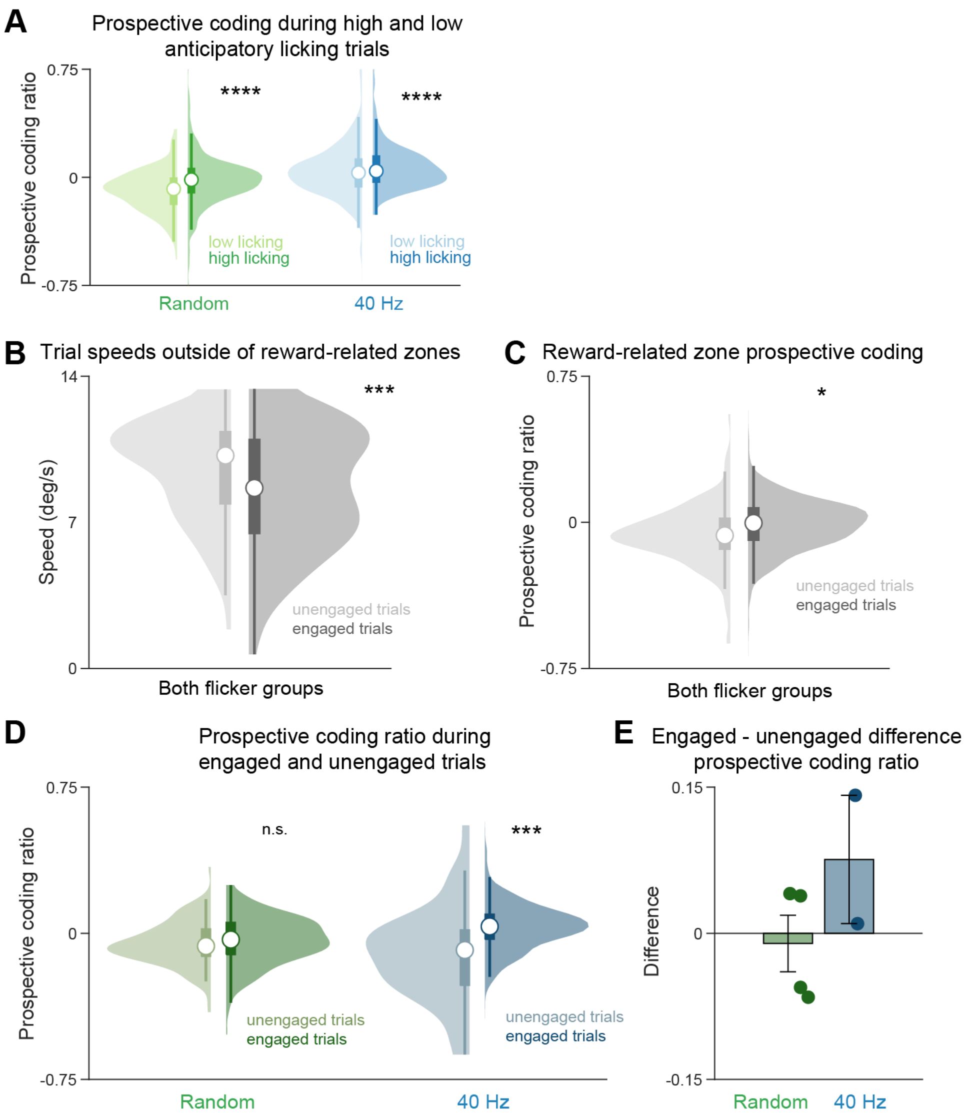

Supplementary Figure 8. Prospective coding is higher on engaged trials after 40 Hz flicker.

A. After 40 Hz and Random flicker, prospective coding was higher on trials with high anticipatory licking than low anticipatory licking. Prospective coding ratio for trials in the top half (high licks, dark colors) and bottom half (low licks, light colors) of anticipatory lick count. Random, low licks n = 354 trials, high licks = 583 trials, from 8 mice; P = 1.24e-06****, LME. 40 Hz, low licks n = 517 trials, high licks n = 290 trials, from 6 mice; P=2.48e-07****, LME.

B. Distributions of trial speeds during unengaged (light grey) and engaged correct (dark grey) trials from Random and 40 Hz flicker groups combined. During unengaged trials, where the animal runs through the environment but does not pause to lick, trial speeds were significantly faster than trials where the animal was engaged in the task and correctly licked in the reward zone. Only data from recording days with both unengaged and engaged trials were included. Unengaged n = 165 trials from 5 mice, engaged n = 455 trials from 5 mice, P = 0.0007***, LME. See Supplementary Table 3 for statistical details.

C. Propsective coding ratios did not differ significantly between unengaged (light grey) and engaged correct (dark grey) trials when animals from both flicker groups were considered together. Ratios

were calculated in the reward-related areas of the track, as in Figure 3. Unengaged n = 165 trials from 5 mice, engaged n = 455 trials from 5 mice, P = 0.023*, LME.

D. Prospective coding ratios were higher after 40 Hz flicker (blue, right) during correct trials when the animal was engaged in the virtual reality task than unengaged trials. After random flicker, prospective coding ratios were not signficaintly different on unengaged and engaged correct trials. Random, unengaged n = 128 trials, engaged 292 trials, from 3 mice, P = 0.49, n.s., LME; 40 Hz, unengaged n = 37 trials, engaged n = 163 trials, from 2 mice, P = 0.0001***, LME.

E. Comparing prospective coding on engaged and unengaged trials within each recording day, prospective coding ratios were higher during engaged, correct trials (difference values greater than 0) after 40 Hz flicker. After Random flicker, prospective coding ratio were higher during engaged correct trials on half the days and lower on half the days. Random, n = 4 days from 3 mice, 40 Hz, n = 2 days from 2 mice. Mean +/- SEM. Each dot is a recording day.

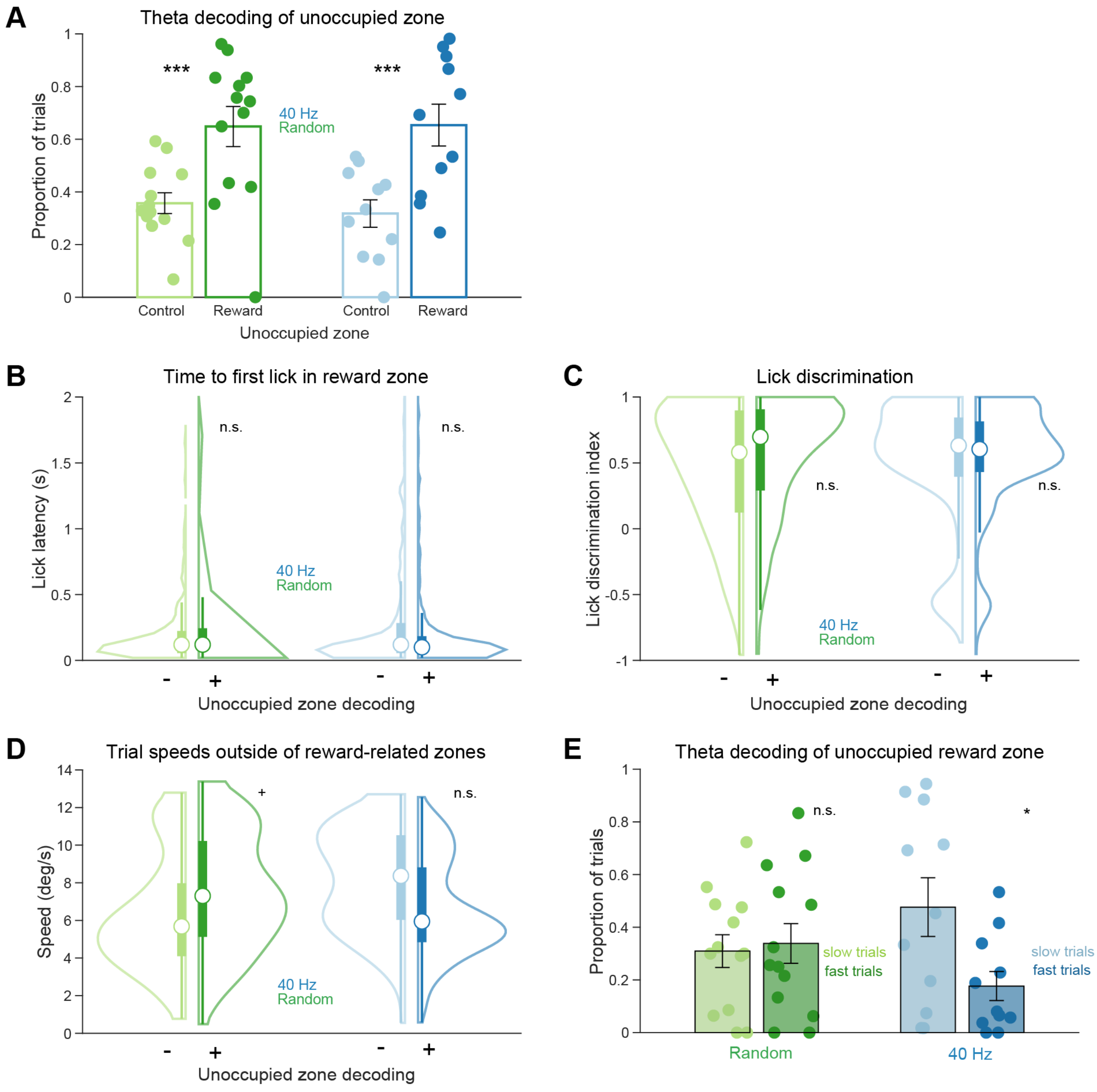

Supplementary Figure 9. Representation of the unoccupied reward zone during theta.

- **A.** After both 40 Hz and Random flicker, most trials showed significant decoding of the unoccupied reward zone during theta (left). A larger proportion of trials showed decoding of the unoccupied reward zone, as compared to the unoccupied control zone (right). Random, unoccupied control zone, n = 13 days, unoccupied reward zone, n = 13 days; from 8 mice; P = 0.0014***, LME. 40 Hz, unoccupied control zone, n = 11 days, unoccupied reward zone, n = 11 days; from 6 mice; P = 0.0019***, LME. Mean ± SEM across recording days. Each dot is a recording day. See Supplementary Table 3 for statistical details.
- **B.** Lick latency, the time to the animal's first lick in the reward zones of the track, was not significantly different between trials with or without theta decoding of the unoccupied reward zone. Random, no unoccupied zone decoding, n = 280 trials, unoccupied zone decoding, n = 606 trials; from 8 mice; P = 0.69, n.s., LME. 40 Hz, no unoccupied zone decoding, n = 346 trials, unoccupied zone decoding, n = 474 trials; from 6 mice; P = 0.21, LME.

C. Lick discrimination, the proportion of licking occuring in the rewarded areas of the track versus other areas, was not significantly different between trials with or without theta decoding unoccupied reward zone. Random, no unoccupied zone decoding, n = 280 trials, unoccupied zone decoding, n = 606 trials; from 8 mice; P = 0.15, n.s., LME. 40 Hz, no unoccupied zone decoding, n = 346 trials, unoccupied zone decoding, n = 474 trials; from 6 mice; P = 0.21, LME.

D. Trial speed, calculated outside of the reward-related areas of the track, was not significantly different between trials with or without theta decoding of the unoccupied reward zone. Random, no unoccupied zone decoding, n = 280 trials, unoccupied zone decoding, n = 606 trials; from 8 mice; P = 0.06+, LME. 40 Hz, no unoccupied zone decoding, n = 346 trials, unoccupied zone decoding, n = 474 trials; from 6 mice; P = 0.87, LME.

E. Proportion of slow or fast trials that contain theta decoding of the unoccupied reward zone. After 40 Hz flicker, slow trials contain a significantly larger proportion of trials that have significant decoding of the other reward zone. Random, proportion of slow trials, n = 13 days, proportion of fast trials, n = 13 days; from 8 mice; P = 0.98, n.s., LME. 40 Hz, proportion of slow trials, n = 11 days, proportion of fast trials n = 11 days; from 6 mice; P = 0.028*, LME. Mean $\pm$ SEM across recording days. Each dot is a recording day.

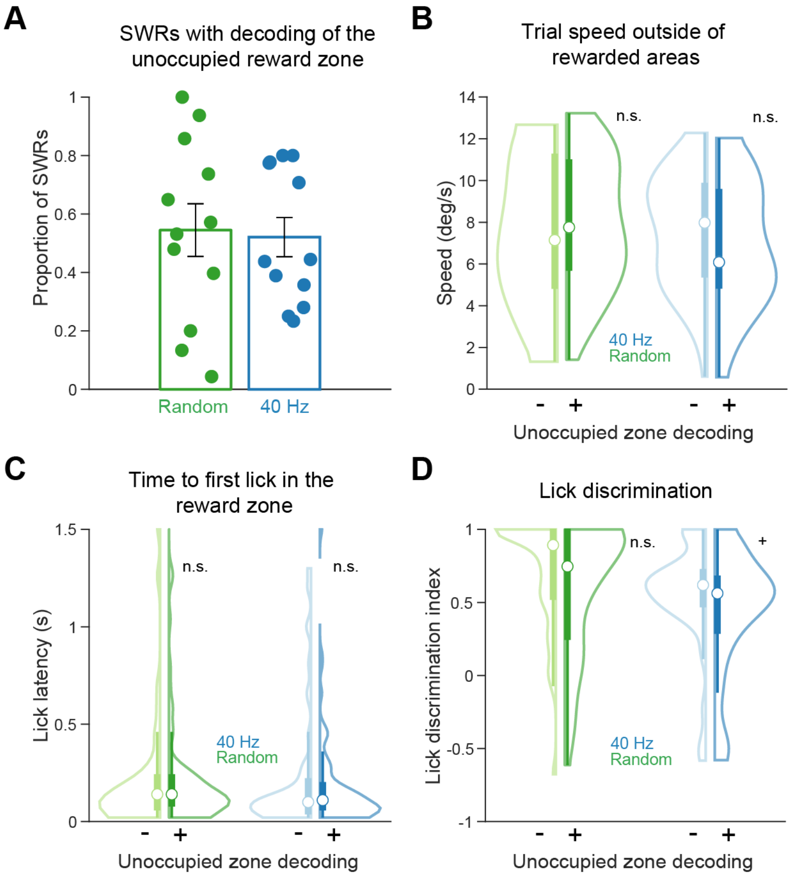

Supplementary Figure 10. Representation of the unoccupied reward zone during SWRs.

- **A.** Proportion of SWRs that contain significant position information representing the unoccupied reward zone. Only SWRs occurring in the reward zones were included for analysis. Random, n = 12 days from 7 mice; 40 Hz, n = 12 days from 7 mice. Mean ± SEM across recording days. Each dot is a recording day. See Supplementary Table 3 for statistical details.
- **B.** Trial speed, calculated outside of the reward-related areas of the track, was not significantly different between trials with (+) or without (-) decoding of the unoccupied reward zone during SWRs. Random, no unoccupied zone decoding (-), n = 42 trials, unoccupied zone decoding (+), n = 94 trials; P = 0.40, n.s., LME. 40 Hz, no unoccupied zone decoding (-), n = 84 trials, unoccupied zone decoding (+), n = 76 trials; P = 0.12, n.s., LME.
- **C.** Lick latency, the time to the animal's first lick in the reward zones of the track, was not significantly different between trials with or without decoding of the unoccupied reward zone during SWRs. Random trials, no unoccupied zone decoding, 1 data point above 1.5, and Random trials with unoccupied zone decoding, 2 data points above 1.5, are excluded for visualization purposes. Random, no unoccupied zone decoding (-), n = 42 trials, unoccupied zone decoding (+), n = 94 trials; P = 0.68, n.s., LME. 40 Hz, no unoccupied zone decoding (-), n = 84 trials, unoccupied zone decoding (+), n = 76 trials; P = 0.25, n.s., LME.
- **D.** Lick discrimination, the proportion of licking occuring in the rewarded areas of the track versus other areas, was not significantly different between trials with or without decoding of unoccupied reward zone during SWRs. Random, no unoccupied zone decoding (-), n = 42 trials, unoccupied zone decoding (+), n = 94 trials; P = 0.41, n.s., LME. 40 Hz, no unoccupied zone decoding (-), n = 84 trials, unoccupied zone decoding (+), n = 76 trials; P = 0.07+, LME.

AnimalID	Flicker Group	Recorded Cells	CA1 cells	CA3 cells	Putative Pyramidal Cells	Putative Interneurons	Place cells	# Trials	# SWRs
28	40 Hz	171	103	68	128	9	102	145	41
29	Random	182	91	91	147	12	107	154	133
30	40 Hz	140	79	61	105	12	81	48	1224
31	Random	92	22	70	49	10	21	107	725
32	40 Hz	199	106	93	146	17	98	80	294
35	40 Hz	93	38	55	75	4	49	20	131
36	Random	164	80	84	128	9	69	142	130
37	Random	188	86	102	117	20	66	115	318
39	40 Hz	117	27	90	78	7	42	175	52
40	Random	139	88	51	109	7	46	140	76
41	40 Hz	218	78	140	170	5	79	25	156
42	Random	180	95	85	127	9	52	111	110
45	40 Hz	196	118	78	150	11	74	115	12
46	Random	136	76	60	84	10	46	133	322
47	40 Hz	109	63	46	82	7	58	292	239
48	Random	141	76	65	104	5	64	95	239

Supplementary Table 1. Recording information.

Figure	Panel	Analysis	Linear Mixed-Effects			Group	N	Unit	# Mice	Mean	SEM	Median	1st Quartile	3rd Quartile
			P-value	Notation	F-statistic									
2	C	PYR slow gamma PPC flicker periods	0.433	ns	$F_{1,13.89} = 0.65$	random	320	cells	8	5.48E-05	3.06E-06	2.96E-05	1.65E-06	7.61E-05
						40 Hz	387	cells	8	8.91E-05	4.53E-06	6.17E-05	1.63E-05	1.18E-04
	C	PYR slow gamma PPC VR task	0.024	*	$F_{1,13.90} = 6.42$	random	347	cells	8	3.99E-05	2.14E-06	2.26E-05	3.83E-06	5.30E-05
						40 Hz	335	cells	8	1.13E-04	6.19E-06	6.92E-05	1.74E-05	1.44E-04
	D	IN slow gamma PPC flicker periods	0.097	+	$F_{1,11.76} = 3.26$	random	46	cells	8	8.46E-05	1.25E-05	5.37E-05	1.22E-05	1.23E-04
						40 Hz	50	cells	8	1.79E-04	2.53E-05	1.08E-04	4.21E-05	1.96E-04
	D	IN slow gamma PPC VR task	0.021	*	$F_{1,8.88} = 7.81$	random	49	cells	8	8.83E-05	1.26E-05	5.74E-05	2.43E-05	1.25E-04
						40 Hz	45	cells	8	2.03E-04	3.03E-05	1.14E-04	6.10E-05	2.57E-04
3	E	theta prospective coding ratio	0.003	**	$F_{1,10.63} = 15.15$	random	13	days	8	-0.033	0.017	-0.019	-0.062	-0.004
						40 Hz	10	days	6	0.052	0.016	0.054	0.025	0.090
	F	prospective coding slow trials	0.005	**	$F_{1,11.08} = 12.57$	random	480	trials	8	-0.039	0.010	-0.030	-0.122	0.048
						40 Hz	389	trials	6	0.050	0.009	0.035	-0.059	0.127
	F	prospective coding fast trials	0.0007	***	$F_{1,7.99} = 28.56$	random	454	trials	8	-0.061	0.008	-0.062	-0.146	0.032
						40 Hz	418	trials	6	0.064	0.009	0.043	-0.042	0.147
	H	reward-related place cells	0.783	ns	$F_{1,11.80} = 0.079$	random	13	days	8	0.169	0.027	0.214	0.062	0.236
						40 Hz	10	days	6	0.166	0.020	0.160	0.107	0.219
4	C	SWR prospective coding ratio	0.047	*	$F_{1,11.30} = 4.95$	random	507	SWRs	7	0.047	0.012	0.041	-0.109	0.208
						40 Hz	378	SWRs	7	0.200	0.016	0.212	-0.001	0.433
	E	reward zone prospective SWRs	0.033	*	$F_{1,11.22} = 5.88$	random	12	days	7	0.387	0.061	0.405	0.203	0.568
						40 Hz	12	days	7	0.606	0.056	0.633	0.5	0.75
5	C	trial speed slow trials	0.214	ns	$F_{1,11.77} = 1.72$	random	483	trials	8	4.495	0.064	4.692	3.538	5.684
						40 Hz	389	trials	6	5.028	0.055	5.243	4.449	5.804
	C	trial speed fast trials	0.993	ns	$F_{1,12.12} = 9e\text{-}05$	random	454	trials	8	9.600	0.093	9.516	7.693	11.428
						40 Hz	418	trials	6	9.489	0.080	9.627	8.227	10.828
	E	prospective coding random	0.009	**	$F_{1,929.14} = 6.93$	slow trials	483	trials	8	0.047	0.008	0.013	-0.043	0.113
						fast trials	454	trials	8	0.051	0.007	0.041	-0.037	0.132
	E	prospective coding 40 Hz	1.10E-09	****	$F_{1,490.72} = 38.58$	slow trials	389	trials	6	-0.007	0.007	-0.012	-0.083	0.066
						fast trials	418	trials	6	0.109	0.008	0.092	0.003	0.209
	F	time to next reward zone random	<2e-16	****	$F_{1,728.93} = 185.27$	slow trials	386	trials	8	35.16	0.696	31.94	28.06	37.56
						fast trials	417	trials	8	18.55	0.205	17.46	15.5	20.92
	F	time to next reward zone 40 Hz	<2e-16	****	$F_{1,415.98} = 173.74$	slow trials	475	trials	6	43.93	1.29	37.3	29.92	47.8
						fast trials	454	trials	6	19.73	0.26	18.11	15.46	22.8

Supplementary Table 2. Statistical information for main figures. Pyramidal cells (PYR); Interneurons (IN).

Figure	Panel	Analysis	P-value	Notation	F-statistic	Group	N	Unit	# Mice	Mean	SEM	Median	1st Quartile	3rd Quartile
S1	G	speed	0.51	ns	$F_{1,13.96} = 0.46$	random	15	days	8	3.784	0.347	3.764	2.818	4.818
						40 Hz	15	days	8	3.455	0.372	2.746	2.638	4.975
	I	lick latency	0.74	ns	$F_{1,13.86} = 0.12$	random	15	days	8	0.464	0.181	0.251	0.149	0.361
						40 Hz	15	days	8	0.578	0.207	0.209	0.158	0.555
S4	C	PYR theta PPC flicker periods	0.19	ns	$F_{1,13.19} = 1.91$	random	320	cells	8	2.07E-03	1.16E-04	2.00E-03	1.42E-03	2.58E-03
						40 Hz	387	cells	8	1.62E-03	8.21E-05	1.61E-03	8.18E-04	2.29E-03
	C	PYR theta PPC VR task	0.29	ns	$F_{1,14.00} = 1.21$	random	347	cells	8	1.00E-03	5.39E-05	7.41E-04	3.50E-04	1.36E-03
						40 Hz	335	cells	8	1.66E-03	9.08E-05	1.13E-03	5.86E-04	2.00E-03
	D	IN theta PPC flicker periods	0.36	ns	$F_{1,8.93} = 0.93$	random	46	cells	8	2.52E-03	3.72E-04	2.47E-03	1.24E-03	3.61E-03
						40 Hz	50	cells	8	2.86E-03	4.04E-04	2.63E-03	1.87E-03	3.54E-03
	D	IN theta PPC VR task	0.12	ns	$F_{1,13.23} = 2.73$	random	49	cells	8	1.73E-03	2.47E-04	0.001384	7.06E-04	2.02E-03
						40 Hz	50	cells	8	2.68E-03	3.79E-04	2.10E-03	1.30E-03	3.18E-03
	E	PYR medium 40 Hz PPC flicker periods	0.90	ns	$F_{1,13.29} = 0.015$	random	320	cells	8	1.40E-05	7.85E-07	4.89E-06	-5.39E-06	2.43E-05
						40 Hz	387	cells	8	1.64E-05	8.35E-07	7.74E-06	-3.00E-07	2.64E-05
	E	PYR medium 40 Hz PPC VR task	0.32	ns	$F_{1,12.99} = 1.09$	random	347	cells	8	8.57E-06	4.60E-07	1.61E-07	-5.35E-06	1.79E-05
						40 Hz	335	cells	8	1.53E-05	8.37E-07	4.25E-06	-4.60E-06	2.27E-05
	F	IN medium 40 Hz PPC flicker periods	0.21	ns	$F_{1,11.52} = 1.75$	random	46	cells	8	2.67E-05	3.93E-06	1.39E-05	5.62E-06	3.70E-05
						40 Hz	50	cells	8	4.52E-05	6.39E-06	2.31E-05	8.28E-06	5.20E-05
	F	IN medium 40 Hz PPC VR task	0.11	ns	$F_{1,8.88} = 3.10$	random	49	cells	8	2.83E-05	4.05E-06	1.87E-05	6.87E-06	4.17E-05
						40 Hz	45	cells	8	4.68E-05	6.98E-06	2.28E-05	8.68E-06	6.96E-05
S6	B	spatial information	0.93	ns	$F_{1,13.98} = 0.01$	random	471	place cells	8	0.253	0.010	0.197	0.107	0.318
						40 Hz	583	place cells	8	0.244	0.010	0.177	0.081	0.318
	C	sparsity	0.69	ns	$F_{1,13.98} = 0.17$	random	471	place cells	8	0.183	0.008	0.122	0.048	0.266
						40 Hz	583	place cells	8	0.190	0.008	0.126	0.048	0.282
	D	peak position	0.68	ns	$F_{1,13.58} = 0.18$	random	471	place cells	8	9.013	2.369	6.000	-30.000	54.000
						40 Hz	583	place cells	8	11.027	2.115	12.000	-27.000	51.000
	E	peak firing rate	0.86	ns	$F_{1,13.58} = 0.03$	random	471	place cells	8	3.731	0.134	2.772	1.523	4.950
						40 Hz	583	place cells	8	3.646	0.115	2.748	1.533	5.165
	F	mean firing rate	0.85	ns	$F_{1,13.68} = 0.04$	random	471	place cells	8	2.127	0.095	1.367	0.652	2.854
						40 Hz	583	place cells	8	2.103	0.085	1.293	0.640	2.871
	G	theta sequence quadrant ratio	0.3702	ns	$F_{1,10.63} = 0.88$	random	13	days	8	0.017	0.003	0.013	0.009	0.026
						40 Hz	10	days	6	0.023	0.007	0.020	0.009	0.025
	I	prospective coding ratio	0.0007	***	$F_{1,11.92} = 20.82$	random	934	trials	8	-0.049	0.006	-0.048	-0.134	0.045
						40 Hz	807	trials	6	0.057	0.006	0.037	-0.049	0.137
	J	RRZ vs. control prospective coding ratio	0.0089	**	$F_{1,1856.02} = 6.85$	random; reward zone	934	trials	8	-0.049	0.006	-0.048	-0.134	0.045
						random; control zone	931	trials	8	-0.029	0.005	-0.027	-0.123	0.062
	J	RRZ vs. control prospective coding ratio	1.4E-13	****	$F_{1,1599.14} = 55.70$	40 Hz; reward zone	807	trials	6	0.057	0.006	0.037	-0.049	0.137
						40 Hz; control zone	799	trials	6	-0.028	0.009	-0.029	-0.149	0.101
S7	A	all SWR count	0.46	ns	$F_{1,11.76} = 0.59$	random	12	days	7	95.42	19.43	95.50	31.50	126.50
						40 Hz	12	days	7	165.42	69.82	45.50	20.50	229.00
	B	SWR prospective coding ratio	0.21	ns	$F_{1,11.87} = 1.77$	random	1145	SWRs	7	0.082	0.008	0.073	-0.095	0.261
						40 Hz	1989	SWRs	7	0.176	0.006	0.188	0.013	0.355
	C	prospective SWRs	0.12	ns	$F_{1,11.27} = 2.88$	random	12	days	7	0.417	0.066	0.408	0.230	0.589
						40 Hz	12	days	7	0.587	0.060	0.629	0.461	0.724
	D	SWR duration	0.75	ns	$F_{1,23.55} = 0.10$	random	572	SWRs	8	0.101	0.002	0.093	0.072	0.122
						40 Hz	475	SWRs	8	0.101	0.002	0.091	0.071	0.123
	E	SWR power	0.41	ns	$F_{1,24.17} = 0.71$	random	572	SWRs	8	5.101	0.092	4.307	3.527	5.843
						40 Hz	475	SWRs	8	5.114	0.128	4.208	3.391	5.684
	F	SWR abundance	0.45	ns	$F_{1,10.72} = 0.62$	random	13	days	8	0.027	0.004	0.023	0.019	0.041
						40 Hz	9	days	6	0.034	0.008	0.027	0.020	0.048
	G	activation probability	0.78	ns	$F_{1,11.78} = 0.084$	random	341	place cells	7	0.388	0.014	0.333	0.192	0.547
						40 Hz	426	place cells	7	0.370	0.012	0.333	0.146	0.559
	H	coactivation probability	0.89	ns	$F_{1,12.00} = 0.018$	random	6519	place cell pairs	7	0.182	0.002	0.123	0.053	0.250
						40 Hz	9641	place cell pairs	7	0.180	0.002	0.111	0.049	0.250
S8	A	propspective coding ratio by anticipatory licking	1.2E-06	****	$F_{1,920.43} = 23.83$	random; low licking	346	trials	8	-0.091	0.008	-0.080	-0.187	-0.003
						random; high licking	588	trials	8	-0.025	0.009	-0.016	-0.108	0.063
	A	propspective coding ratio by anticipatory licking	2.5E-07	****	$F_{1,729.03} = 27.13$	40 Hz; low licking	517	trials	6	0.048	0.009	0.033	-0.066	0.129
						40 Hz; high licking	290	trials	6	0.074	0.010	0.044	-0.034	0.150
	B	engaged vs. unengaged trial speeds	0.0007	***	$F_{1,615.97} = 11.67$	unengaged trials	165	trials	5	9.492	0.187	10.201	7.879	11.350
						engaged trials	455	trials	5	8.517	0.135	8.644	6.457	10.975
	C	engaged vs. unengaged prospective coding ratio	0.02	*	$F_{1,615.04} = 5.15$	unengaged trials	165	trials	5	-0.066	0.012	-0.067	-0.139	0.022
						engaged trials	455	trials	5	-0.012	0.007	-0.004	-0.093	0.075
	D	engaged vs. unengaged prospective coding ratio	0.49	ns	$F_{1,416.84} = 0.48$	random; unengaged trials	128	trials	3	-0.055	0.010	-0.065	-0.118	0.023
						random; engaged trials	292	trials	3	-0.036	0.007	-0.032	-0.109	0.057
	D	engaged vs. unengaged prospective coding ratio	0.0001	***	$F_{1,197.95} = 15.68$	40 Hz; unengaged trials	37	trials	2	-0.105	0.040	-0.086	-0.267	0.018
						40 Hz; engaged trials	163	trials	2	0.032	0.012	0.036	-0.031	0.099
S9	A	unoccupied zone decoding proportion of trials	0.0001	***	$F_{1,17.04} = 23.97$	random; reward zone	13	days	8	0.357	0.040	0.329	0.291	0.468
						random; control zone	13	days	8	0.648	0.076	0.744	0.430	0.833
	A	unoccupied zone decoding proportion of trials	0.0002	***	$F_{1,15.02} = 24.03$	40 Hz; reward zone	11	days	6	0.318	0.052	0.333	0.170	0.460
						40 Hz; control zone	11	days	6	0.653	0.079	0.692	0.411	0.902
	B	unoccupied zone decoding lick latency	0.69	ns	$F_{1,505.48} = 0.16$	random; no unoccupied zone decoding	280	trials	8	0.219	0.024	0.120	0.060	0.220
						random; unoccupied zone decoding	606	trials	8	0.362	0.059	0.120	0.080	0.240
	B	unoccupied zone decoding lick latency	0.21	ns	$F_{1,817.53} = 1.55$	40 Hz; no unoccupied zone decoding	346	trials	6	0.298	0.026	0.120	0.060	0.280
						40 Hz; unoccupied zone decoding	474	trials	6	0.211	0.017	0.100	0.060	0.180
	C	unoccupied zone decoding lick discrimination	0.15	ns	$F_{1,880.41} = 2.06$	random; no unoccupied zone decoding	280	trials	8	0.468	0.028	0.581	0.127	0.894
						random; unoccupied zone decoding	606	trials	8	0.553	0.018	0.698	0.295	0.903
	C	unoccupied zone decoding lick discrimination	0.21	ns	$F_{1,817.49} = 1.58$	40 Hz; no unoccupied zone decoding	346	trials	6	0.504	0.026	0.632	0.399	0.840
						40 Hz; unoccupied zone decoding	474	trials	6	0.547	0.019	0.604	0.434	0.810
	D	unoccupied zone decoding trial speeds	0.07	+	$F_{1,880.96} = 3.39$	random; no unoccupied zone decoding	280	trials	8	6.250	0.179	5.681	4.125	7.949
						random; unoccupied zone decoding	606	trials	8	7.488	0.125	7.301	5.155	10.194
	D	unoccupied zone decoding trial speeds	0.87	ns	$F_{1,814.94} = 0.026$	40 Hz; no unoccupied zone decoding	346	trials	6	8.139	0.142	8.368	6.060	10.502
						40 Hz; unoccupied zone decoding	474	trials	6	6.585	0.119	5.945	4.871	8.793
	E	unoccupied zone decoding by median trial speed	0.77	ns	$F_{1,17.49} = 0.09$	Random; slow trials	13	days	8	0.309	0.062	0.300	0.080	0.479
						Random; fast trials	13	days	8	0.339	0.075	0.256	0.116	0.559
	E	unoccupied zone decoding by median trial speed	0.03	*	$F_{1,15.19} = 5.82$	40 Hz; slow trials	11	days	6	0.476	0.111	0.453	0.104	0.843
						40 Hz; fast trials	11	days	6	0.177	0.055	0.080	0.042	0.311
S10	B	SWR unoccupied zone decoding trial speed	0.40	ns	$F_{1,128.91} = 0.72$	random; no unoccupied zone decoding	42	trials	7	7.572	0.543	7.143	4.833	11.256
						random; unoccupied zone decoding	94	trials	7	7.954	0.314	7.756	5.702	10.987
	B	SWR unoccupied zone decoding trial speed	0.12	ns	$F_{1,154.59} = 2.51$	40 Hz; no unoccupied zone decoding	84	trials	7	7.594	0.292	7.979	5.379	9.861
						40 Hz; unoccupied zone decoding	76	trials	7	6.828	0.338	6.084	4.844	9.567
	C	SWR unoccupied zone decoding lick latency	0.68	ns	$F_{1,134.0} = 0.17$	random; no unoccupied zone decoding	42	trials	7	0.285	0.079	0.140	0.060	0.240
						random; unoccupied zone decoding	94	trials	7	0.256	0.039	0.140	0.080	0.240
	C	SWR unoccupied zone decoding lick latency	0.25	ns	$F_{1,155.79} = 1.33$	40 Hz; no unoccupied zone decoding	84	trials	7	0.234	0.035	0.100	0.040	0.220
						40 Hz; unoccupied zone decoding	76	trials	7	0.204	0.033	0.110	0.060	0.200
	D	SWR unoccupied zone decoding lick	0.41	ns	$F_{1,129.26} = 0.69$	random; no unoccupied zone decoding	42	trials	7	0.714	0.060	0.893	0.522	1.000
						random; unoccupied zone decoding	94	trials	7	0.592	0.047	0.747	0.247	1.000
	D	SWR unoccupied zone decoding lick	0.07	+	$F_{1,155.86} = 3.34$	40 Hz; no unoccupied zone decoding	84	trials	7	0.550	0.039	0.619	0.471	0.726
						40 Hz; unoccupied zone decoding	76	trials	7	0.434	0.049	0.562	0.289	0.680

Supplementary Table 3. Statistical information for supplemental figures.

Figure	Panel	Analysis	Significant Frequency Points (p < 0.05, unpaired t-test without correction)
**S3**	**A**	WPLI VR	14.1602, 14.6484, 15.1367, 15.625, 16.1133, 16.6016, 17.0898, 17.5781, 18.0664, 18.5547, 19.043, 23.9258, 26.8555, 27.3438, 28.8086, 29.2969, 29.7852, 30.2734, 30.7617, 31.7383, 32.7148, 34.1797, 34.668, 35.1562, 36.1328, 36.6211
**S3**	**A**	WPLI Flicker periods	10.2539, 10.7422, 11.2305, 11.7188, 12.207, 12.6953, 15.1367, 15.625, 16.1133, 16.6016, 17.0898, 17.5781, 18.0664, 18.5547, 19.043, 19.5312, 20.0195, 20.5078, 20.9961, 21.9727, 22.4609, 24.9023, 35.6445, 39.5508, 40.0391, 119.6289
**S3**	**B**	CA1 PSD VR	6.8359, 24.9023, 26.8555, 27.3438
**S3**	**B**	CA1 PSD Flicker periods	18.5547, 21.9727, 22.4609, 24.9023, 25.3906, 26.3672, 26.8555, 39.5508, 40.0391, 79.5898, 80.0781, 119.6289
**S3**	**C**	CA3 PSD VR	56.1523, 59.5703, 60.0586, 60.5469, 61.5234, 62.0117, 63.4766, 63.9648, 64.4531, 64.9414, 65.4297, 65.918, 66.8945, 67.3828, 67.8711, 68.3594, 68.8477, 69.8242, 70.3125, 70.8008, 71.2891, 71.7773, 72.7539, 74.2188, 74.707, 75.6836, 77.1484, 79.1016
**S3**	**C**	CA3 PSD Flicker periods	5.8594, 13.6719, 14.1602, 39.5508, 40.0391, 52.7344, 55.1758, 56.1523, 56.6406, 57.1289, 57.6172, 58.1055, 58.5938, 59.082, 59.5703, 60.0586, 60.5469, 61.0352, 61.5234, 62.0117, 62.5, 62.9883, 63.4766, 63.9648, 64.4531, 64.9414, 65.4297, 65.918, 66.4062, 66.8945, 67.3828, 67.8711, 68.3594, 68.8477, 69.3359, 69.8242, 70.3125, 70.8008, 71.2891, 71.7773, 72.2656, 72.7539, 73.2422, 73.7305, 74.2188, 74.707, 75.1953, 75.6836, 76.1719, 76.6602, 77.1484, 77.6367, 78.125, 78.6133, 79.1016, 79.5898, 80.0781, 81.0547, 81.543, 82.0312, 82.5195, 83.0078, 83.4961, 83.9844, 84.4727, 84.9609, 85.4492, 85.9375, 86.4258, 86.9141, 87.4023, 87.8906, 88.3789, 88.8672, 89.3555, 89.8438, 90.332, 90.8203, 91.3086, 91.7969, 92.2852, 92.7734, 93.2617, 94.2383, 94.7266, 95.2148, 95.7031, 96.1914, 96.6797, 97.168, 99.6094, 100.0977, 101.5625, 102.0508, 110.3516

Supplementary Table 4. Statistical information for Supplementary Figure 3. Significant frequency points (indicated by * in Supplementary Figure 3) are listed here. Weighted phase-lag index (WPLI); power spectral density (PSD).

Figure	Analysis	Cells per animal (min-max)	
		Random	40 Hz
2C, left	CA3 Pyramidal Cell slow gamma PPC during flicker periods	8 - 67	28 - 99
2C, right	CA3 Pyramidal Cell slow gamma PPC during VR periods	15 - 79	18 - 101
2D, left	CA3 interneuron slow gamma PPC during flicker periods	3 - 12	1 - 15
2D, right	CA3 interneuron slow gamma PPC during VR periods	2 - 8	1 - 5
3D	Propsective coding ratio over position per day	21 - 107	42 - 102
3E	Reward-related prospective coding ratio per day	21 - 107	42 - 102
3F	Reward-related prospective coding ratio by trial speed	21 - 107	42 - 102
3G	Place cell rate maps	21 - 107	42 - 102
3H	Reward-related place cells	1 - 52	5 - 18
4C	Prospective coding ratio during reward zone SWRs	21 - 107	42 - 102
4D	Decoded position during reward zone SWRs	21 - 107	42 - 102
4E	Proportion of prospective reward zone SWRs	21 - 107	42 - 102
5D	Prospective coding ratio during fast and slow trials	21 - 107	42 - 102
5E	Post-reward prospective coding	21 - 107	42 - 102

Supplementary Table 5. Range of cells per animal included in main figure analyses.

Supplementary Figure	Analysis	Cells per animal (min-max)	
		Random	40 Hz
2B, CA3 PYR	Number of classified putative pyramidal cells	24 - 68	31 - 100
2B, CA3 IN	Number of classified putative interneurons	3 - 12	2 - 15
2B, CA1 PYR	Number of classified putative pyramidal cells	13 - 79	18 - 101
2B, CA1 IN	Number of classified putative interneurons	2 - 8	0 - 5
4C, left	CA3 pyramidal cell theta PPC during flicker periods	8 - 67	28 - 99
4C, right	CA3 pyramidal cell theta PPC during VR periods	23 - 66	22 - 73
4D, left	CA3 interneuron theta PPC during flicker periods	3 - 12	1 - 15
4D, right	CA3 interneuron theta PPC during VR periods	3 - 12	1 - 15
4E, left	CA3 pyramidal cell medium gamma PPC during flicker periods	8 - 67	28 - 99
4E, right	CA3 pyramidal cell medium gamma PPC during VR periods	23 - 66	22 - 73
4F, left	CA3 interneuron medium gamma PPC during flicker periods	3 - 12	1 - 15
4F, right	CA3 interneuron medium gamma PPC during VR periods	3 - 12	1 - 15
6A	Place cells	21 - 107	42 - 102
6B	Place cells, spatial information	21 - 107	42 - 102
6C	Place cells, sparsity	21 - 107	42 - 102
6D	Place cells, peak position	21 - 107	42 - 102
6E	Place cells, peak firing rate	21 - 107	42 - 102
6F	Place cells, mean firing rate	21 - 107	42 - 102
6G	Theta sequence strength	21 - 107	42 - 102
6H	Prospective coding ratio over position per trial	21 - 107	42 - 102
6I	Prospective coding ratio in the reward related zone	21 - 107	42 - 102
6J	Propsective coding ratio in control vs. reward-related zone	21 - 107	42 - 102
6K	Propsective coding ratio during high and low anticipatory licking trials	21 - 107	42 - 102
6L	Decoding of current position	21 - 107	42 - 102
6M	Decoding error of current position	21 - 107	42 - 102
7B	Prospective coding ratio during all position SWRs	21 - 107	42 - 102
7C	Proportion of prospective all position SWRs	21 - 107	42 - 102
7G	Place cell activation	21 - 107	42 - 102
7H	Place cell coactivation	21 - 107	42 - 102
8B	Prospective coding ratio (both groups) on unengaged and engaged trials	51 - 69	22 - 57
8C	Prospective coding ratio during unengaged and engaged trials	51 - 69	22 - 57
8D	Prospective coding ratio difference between engaged and unengaged trials	51 - 69	22 - 57
9A	Unoccupied zone decoding during theta	21 - 107	42 - 102
9E	Unoccupied reward zone decoding by speed	21 - 107	42 - 102
10A	SWRs with unoccupied reward zone decoding	21 - 107	42 - 102

Supplementary Table 6. Range of cells per animal included in supplementary figure analyses.
