# Supplementary material for: 40 Hz sensory stimulation enhances CA3-CA1 coordination and prospective coding during navigation in a mouse model of Alzheimer’s disease
Source: bioRxiv. 2024 Oct 23:2024.10.23.619408. Preprint. [Version 1] doi: 10.1101/2024.10.23.619408 (PMC11526945; doi:10.1101/2024.10.23.619408)
Supplement: Supplement 2 [file NIHPP2024.10.23.619408v1-supplement-2.pdf]

bioRxiv preprint doi: https://doi.org/10.1101/2024.10.23.619408; this version posted October 23, 2024. The copyright holder for this preprint (which was not certified by peer review) is the author/funder, who has granted bioRxiv a license to display the preprint in perpetuity. It is made available under aCC-BY 4.0 International license.

Additional Files

Additional File 1
.mov
A headfixed mouse performs the virtual reality spatial navigation task.
The mouse runs on the spherical treadmill, which controls movement through the virtual reality track. The mouse must lick in specific zones of the track to receive a reward of sweetened condensed milk.
